# Ontogeny, distribution and function of CD38-expressing B lymphocytes in mice

**DOI:** 10.1002/1521-4141(200104)31:4<1261::AID-IMMU1261>3.0.CO;2-H

**Published:** 2001-03-29

**Authors:** Felipe Raúl Donís-Hernández, R Mike E Parkhouse, Leopoldo Santos-Argumedo

**Affiliations:** 1 Department of Molecular Biomedicine, Centro de Investigación y Estudios Avanzados del I.P.N.México D. F., México; 2 Instituto Gulbenkian de CienciaOeiras, Portugal

**Keywords:** Ontogeny, CD38, Peritoneal B Lymphocyte, Proliferation, Distribution

## Abstract

Analysis of expression of CD38, CD45R (B220), IgM and IgD on splenic B lymphocytes from mice of different ages demonstrated CD38 on both immature (B220^+^, BCR^–^) and mature (B220^+^, BCR^+^) B lymphocytes. Similarly, CD38 is expressed as early as B220 on the surface of progenitor B cells in the bone marrow. In spite of expressing of CD38 and IgM, neonatal B cells, in contrast to the adult, failed to proliferate to either anti-CD38 or anti-IgM cross-linking when IL-4 was present. They did, however, respond to LPS and anti-CD40, and by 2 weeks of age they began to respond to anti-CD38 and anti-IgM, reaching adult B cell levels by 4 weeks. Although the distribution of CD38 on adult B cells from most different lymphoid compartments was broadly similar, significantly higher levels of CD38 were expressed on peritoneal B lymphocytes. A detailed analysis, using IgM / IgD ratio and staining with anti-CD5 confirmed that B1 lymphocytes were expressinga high level of CD38. Interestingly, both immature B cells and peritoneal B1 lymphocytes were unresponsive to anti-CD38. However, they were activated by LPS or anti-CD40.

## 1 Introduction

The differentiation of lymphocytes can be studied through the analysis of surface "markers" appearing or disappearing between discrete phases of the process. These molecules have been named differentiation antigens, and many of them have a cluster of differentiation (or CD) number. The precise function of some of these molecules is still obscure, but some of them have been identified as receptors for growth factors, adhesion molecules, growth inhibitory molecules, etc. Clearly, the study of these molecules will make a major contribution to our understanding of ontogeny, developmentand function of the immune system.

The ecto-enzyme CD38 is one such differentiation antigens, whose expression is restricted, but not exclusive to murine B lymphocytes 1. In humans, CD38 has been used as a negative marker for stem cells in bone marrow, to classify malign lymphocytic leukemias (*e. g.* prognosis of human B-CLL), for monitoring HIV infection and progression and as a target for the treatment of myeloma 2–5. The extracellular domain of CD38 catabolizes the conversion of NAD^+^ to cyclic-ADP-ribose (cADPR), nicotinic acid adenine dinucleotide (NAADP), ADP-ribose (ADPR) and nicotinic acid 6, 7. Both cADPR and NAADP are powerful calcium messengers in a wide variety of cells 7, 8. However, the role of these products in the proliferation and differentiation process of either human or murine lymphocytes have not been explored. The question of whether the extracellular enzymatic activity of CD38 is related to a defined function or, for example is a vestigial remain of a previous function, is not resolved.

Anti-CD38 induces a variety of measurable activation events in both B or T lymphocytes: proliferation of B lymphocytes 9, induction of production and secretion of cytokines by T lymphocytes 10, regulation of T-cell activities 11, activation of NK cells and monocytes 12, 13, rescue from apoptosis 9, 14, induction of apoptosis in immature B cell from human bone marrow 15, 16, phosphorylation of different intracellular substrates and modulator of homotypic and heterotypic adhesion 17, 18. Yet the biological role of CD38 and its possible requirements for the enzymatic activity remain controversial and thus, a defined role during B cell differentiation is still lacking. The aim of this work was to study the ontogeny of expression CD38 on lymphocytes of different compartments, in order to better understand the possible role of this molecule during B cell differentiation. The development of responsiveness of total and B1 B lymphocytes to anti-CD38, anti-IgM, CD40L and LPS was also studied. Although both immature and B1 B lymphocytes respond to LPS and anti-CD40, they were unresponsive to anti-IgM and anti-CD38, and so it is possible that CD38 may play an as-yet-undefined role in these two B cell subpopulations. In mature B cells, however, the functional association of stimulation with anti-Ig and anti-CD38 is consistent with the hypothesis that CD38 may use part of the BCR transduction machinery to activate mature B cells.

## 2 Results

### 2.1 Ontogeny of expression of CD38 in the spleen

**Fig. 1:**
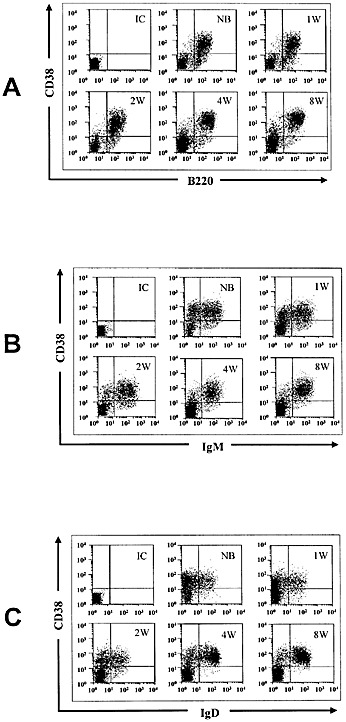
Ontogeny of the expression of CD38 in the spleen. Splenic lymphocytes from BALB / c mice at different ages were stained with B220-FITC (A), anti-IgM-FITC (B) or anti-IgD-FITC (C) and counter-stained with anti-CD38-PE. IC: isotype control staining for the pairs used in each combination; NB: newborn mice; 1 – 8W, 1 – 8 weeks old.

To study the ontogeny of expression of CD38, spleens from BALB / c mice at different ages were analyzed, comparing the expression of three well-known markers (B220, IgM and IgD) with CD38. The results are shown in Fig. 1 for each of the three markers. One of the earliest markers, B220, is present on all B lymphocytes in spleen and, as can be seen in Fig. 1 A, newborn mice express both B220 and CD38. The intensity of their expression increases upon maturation and reaches adult levels at 4 weeks of age. Newborn and 1-week-old mice have a small proportion of B220^+^, CD38^–^ lymphocytes, but this population essentially disappears by 4 weeks of age. As shown in Fig. 1 B CD38 is expressed before IgM but all IgM^+^ cells are also CD38^+^. Thus in newborn and 1-week- old mice there are CD38^+^ B cells not yet expressing IgM. In older mice, the expression of both IgM and CD38 is increased, reaching adult levels at 8 weeks of age. Finally, CD38 is clearly expressed on IgD^–^ cells, with IgD^+^ cells reaching adult levels by 8 weeks (Fig. 1 C).

### 2.2 Expression of CD38 in bone marrow from adult mice

**Fig. 2:**
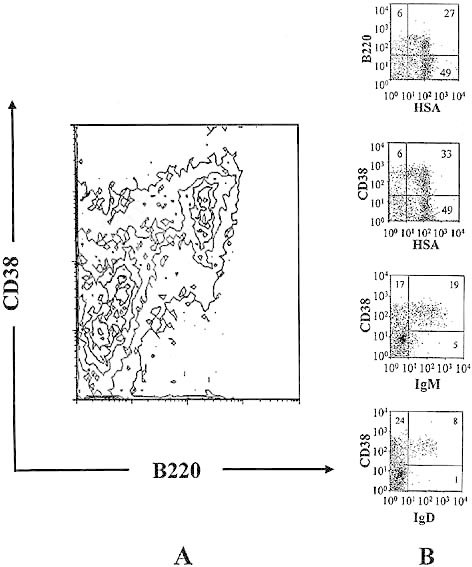
Expression of CD38 in bone marrow from adult mice. Bone marrow cells from 8-week-old BALB / c mice were stained with B220-FITC and anti-CD38-PE for panel A; and anti-HSA-FITC, anti-IgM-FITC or anti-IgD-FITC and then counter-stained with B220-PE or anti-CD38-PE for panel B.

To confirm and extend the results from the spleen, bone marrow cells from adult mice were similarly analyzed. As was previously shown, CD38 and B220 are co-expressed and so CD38 is one of the earliest markers in B cell differentiation. A detailed analysis, using the heat-stable antigen (HSA) 19 to define better the ontogeny of appearance of CD38 on B cells is presented in Fig. 2. CD38 is expressed on pro-B cells, probably at the same time as B220, and prior to expression of first surface IgM, and then IgD.

### 2.3 Immature B lymphocytes fail to proliferate to anti-CD38 stimulation

**Fig. 3:**
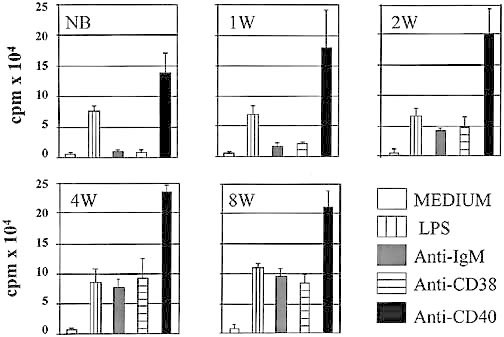
Immature B lymphocytes fail to proliferate to anti-CD38 stimulation. 10^6^-MACS purified splenic B lymphocytes from BALB / c mice at different ages were stimulated with 50 μg / ml LPS, 10 μg / ml anti-mouse IgM (B7.6), 20 μg / ml anti-mouse CD38 (NIM-R5), or 10 μg / ml anti-mouse CD40 (1C10). All cultures contained 10 U / ml of recombinant mouse IL-4. The cultures were incubated for 72 h at 37 °C, 5 % CO_2_ and pulsed with [^3^H] thymidine during the last 4 h of culture. Results are shown as mean cpm plus standard deviation from four wells. This is a representative experiment of five done in the same manner and with similar results.

Immature B cells proliferate to LPS stimulation but fail to proliferate to surface IgM cross-linking 20, 21. Since immature B lymphocytes express CD38 on the surface, we analyzed their responsiveness to stimulation through CD38 in the presence of IL-4. Even though immature B lymphocytes (from newborn and 1-week-old mice) express significant levels of CD38, they failed to proliferate to anti-CD38 and anti-IgM stimulation, but did respond to LPS and anti-CD40. Proliferation to CD38, similar to anti-IgM-induced responses, started in mice from 2 weeks of age and reached adult levels in 4-week-old mice (Fig. 3).

### 2.4 B lymphocytes from C57BL / 6 and C3H / HeN express and proliferate to CD38

**Fig. 4:**
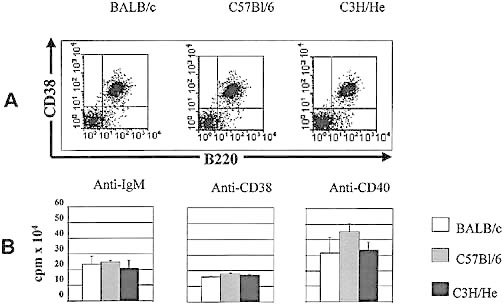
B lymphocytes from three strains of adult mice express and proliferate to anti-CD38 stimulation. Splenic lymphocytes from three strains of adult mice were stained with anti-B220-FITC / anti-CD38-PE (panel A). Purified B lymphocytes from spleen of each strain of mice were stimulated with 10 μg / ml anti-mouse IgM (B7.6), 20 μg / ml anti-mouse CD38 (NIM-R5), or 10 μg / ml anti-mouse CD40 (1C10). All cultures contained 10 U / ml of recombinant mouse IL-4. The cultures were incubated for 72 h at 37 °C, 5 % CO_2_ and pulsed with [^3^H] thymidine during the last 4 h of culture. Results are shown as mean cpm plus standard deviation from 4 wells (panel B). This is a representative experiments of five done in the same manner and with similar results.

A similar analysis was done with two other strains of mice (C57BL / 6 and C3H / HeN) in order to ensure the generality of the results obtained with the BALB / c strain as described above. The FACS analysis of surface marker expression and responsiveness to anti-IgM, anti-CD40 and anti-CD38 were similar in the three strains of mice (Fig. 4). Thus all three strains of mice were equally able to proliferate in response to CD38 cross-linking. We have previously shown proliferative responses of B cells from DBA / 2 9, 129 22 and CBA 23 mice upon stimulation with monoclonal anti-CD38 antibody NIM-R5.

### 2.5 Expression of CD38 on B lymphocytes from different lymphoid compartments

**Fig. 5:**
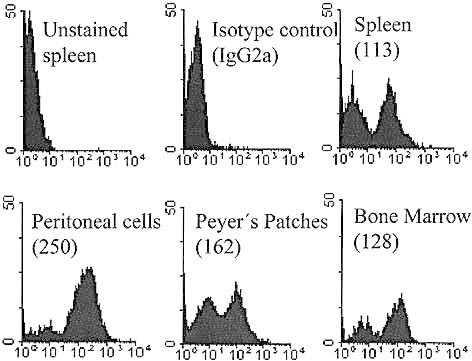
Differential expression of CD38 on B lymphocytes from different lymphoid compartments. Total lymphocytes from spleen, peritoneal cavity, Peyer's patches and bone marrow were stained with FITC-anti-mouse CD38 (NIM-R5). Numbers in parentheses show the mean fluorescence intensity of the CD38^+^ cells.

**Fig. 6:**
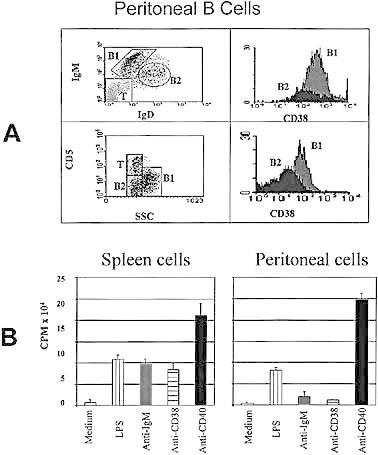
B1 lymphocytes from peritoneal cavity express high levels of CD38 on their surface, but fail to proliferate to CD38 cross-linking. Lymphocytes from the peritoneal cavity were stained with anti-IgD-FITC / anti-IgM-PE, anti-CD38-biotin and then streptavidin-PerCP or with anti-CD38-FITC / anti-CD5-PE (panel A). Purified B lyphocytes from the peritoneal cavity or the spleen were stimulated with 50 μg / ml LPS, 10 μg / ml anti-mouse IgM (B7.6), 20 μg / ml anti-mouse CD38 (NIM-R5), or 10 μg / ml anti-mouse CD40 (1C10). All cultures contained 10 U / ml of recombinant mouse IL-4. The cultures were incubated for 72 h at 37 °C, 5 % CO_2_ and pulsed with [^3^H] thymidine during the last 4 h of culture. Results are shown are mean cpm plus standard deviation from 4 wells (panel B). This is a representative experiments of five done in the same manner and with similar results.

We next analyzed the distribution of CD38 of B cells from Peyer's patches and the peritoneal cavity. The former contains B lymphocytes engaged in local Ig production, whereas the B1 lymphocytes in the latter compartment display a more unique phenotype and function. As can be seen in Fig. 5 and Table 1, lymphocytes from both tissues expressed CD38, but expression of CD38 on peritoneal B1 cells was substantially higher than on any other lymphoid compartment. Peritoneal cavity B cells are resolved into two subpopulations termed B1 and B2, with the B2 cells being similar to normal splenic B lymphocytes whereas the B1 cells display a unique marker phenotype, namely Mac-1^+^, CD23^+^ and high IgM / IgD ratio. Our results clearly demonstrate that the IgM^high^, IgD^low^ B1 cells also have the highest expression of CD38 (Fig. 6 A and Table 1). This result was confirmed by staining with anti-CD5, a marker present on B1 cells but absent on B2 cells. In contrast, the peritoneal B2 cells, characterized by a lower expression of IgM, higher expression of IgD and an absence of CD5, express CD38 at similar levels to B cells in the other B cell compartments analyzed.

**Table 1:** B1 lymphocytes from peritoneal cavity express high levels of surface CD38a)

	MFI	± SD	
Peritoneal B 1 cells	**284**	**± 29**	
Peritoneal B2 cells	132	± 12	p < 0.001*
Bone Marrow	117	± 12	p < 0.001*
Spleen	142	± 28	p < 0.001*
Peyer's Patches	178	± 32	p < 0.001*

a) Results show mean fluorescence intensity (MFI) and standard deviation (SD) of the CD38^+^ cells from five different experiments.

* t-Student's value for comparison between B1 cells with all other populations.

### 2.6 Peritoneal B lymphocytes fail to proliferate in response to anti-CD38 stimulation

Because CD38 is highly expressed by peritoneal B cells, we compared their susceptibility to stimulation with anti-CD38, anti-IgM, anti-CD40 and LPS with the response of splenic B lymphocytes. As can be seen in Fig. 6 B, splenic B cells were able to proliferate to all stimuli. In contrast, peritoneal B lymphocytes did not proliferate in respnse to anti-CD38 and anti-IgM, but were stimulated by anti-CD40 and LPS. The lack of response of peritoneal B cells to CD38 and anti-IgM implies an intrinsic defect in the CD38-IgM signaling pathway of these cells, because they were perfectly able to proliferate to anti-CD40 and LPS stimulation. Even though peritoneal B cells have high levels of CD38 on their surface, they were unable to proliferate to anti-CD38 cross-linking (Fig. 6). These results are reminiscent of the failure of immature B lymphocytes to proliferate to the same stimuli (Fig. 3).

## 3 Discussion

The biological functions described for CD38 include adhesion to endothelial cells 24, 25, production of calcium messengers such as cADPR and NAADP 6–8, and positive or negative regulation of cell activation and proliferation 1, 2, 18. Unfortunately, none of these functions has been confirmed in the analysis of CD38-deficient mice, which have no impairment in either hematopoiesis or lymphopoiesis 22, but may be deficient in the production of antibodies to T-cell dependent antigens.

We found that CD38 is expressed early in ontogeny, probably at the pro-B stage. Splenic B cells from new born mice were mostly B220^+^, CD38^+^; CD38 appears prior to surface IgM and IgD, and its expression increases during development, reaching adult levels at 4 week of age. The expression of murine CD38 is enhanced upon mitogen stimulation, but is reduced on germinal center cells and abrogated when B lymphocytes differentiate into plasma cells 9.

Despite the presence of surface IgM or CD38, immature B lymphocytes do not respond to stimulation via antibodies to either of these two molecules, suggesting that the machinery to transduce signals through CD38 and IgM is deficient. This functional association between IgM and CD38 was first suggested by experiments with B lymphocytes from XID mice 23. It was further extended by the lack of response of B lymphocytes from tolerant mice 26 and through genetic analysis of A.20 lymphoma B cells transfected with genes encoding for BCR and CD38 molecules 27. The general conclusion is that CD38 and IgM are integrated into a common pathway, with some elements of the transduction machinery of surface IgM such as BTK, being used by CD38 23, 28. This may explain why the IgM^+^ CD38^+^ immature B lymphocytes, are unable to transduce a proliferative signal via CD38 or IgM. However, these results still leave a question mark on the role of CD38, a molecule normally present early in ontogeny of B cells, but dispensable for generation of new B lymphocytes in the CD38-deficient mice. Thus CD38 may well have a still unknown role in controlling the development of B lymphocytes, and it is possible that this function is obscured by as yet unknown redundant molecules that compensate the CD38-deficient mice.

An interesting observation is the high expression of CD38 on B1 lymphocytes from the peritoneal cavity. The functional role of the molecule in this population is obscure as these lymphocytes do not proliferate to stimulation with anti-CD38 (or anti-IgM), reinforcing the hypothesis that the CD38 and IgM signal transduction machineries are linked. This result correlates with the observation with XID-B lymphocytes and tolerant B cells which also have CD38 on their surfaces, are unable to proliferate to either anti-IgM or anti-CD38.

One of the most enigmatic questions on CD38 is the role of its enzymatic activity in catalyzing the conversion of NAD^+^ to cADPR (a potent calcium messenger) and ADPR (whose role has not been established, but may have inhibitory functions) 6. The fact that both metabolites are generated extracellularly is impossible to reconcile with a role in intracellular signal transduction, and it has therefore been suggested that cADRP functions exogenously in stimulated B cells 6. It is also possible that this enzyme activity is unrelated to the demonstrated mitogenic activity of CD38 in antibody-mediated signal transduction, as mutants of CD38 without enzymatic activity can still be activated via anti-CD38 29. The possible role of the CD38 ribosyl transferase activity 30 and the nicotinamide metabolic release resulting from the breakdown of NAD^+^, therefore remain to be explored and understood. It is perhaps relevant that nicotinamide has recently been shown to regulate the expression of adhesion molecules and cellular differentiation 31.

An important effect of stimulation via CD38 is the phosphorylation of different intracellular substrates 17, 27, 32–34, a feature, that may be totally independent of the ecto-enzymatic activity of CD38. Thus, CD38 may function as a bi- or multifunctional protein, whose activity could be modulated by different ligands and / or substrates. The search for such ligands is a priority in order to understand the biological role of this molecule. In humans, CD31 has been described as one of such ligands 25, but, this has yet to be confirmed in mice. It is also possible that CD38 may have a different role in immature B *vs.* mature B lymphocytes, and the same may be postulated for B1 *vs.* B2 cells.

## 4 Materials and methods

### 4.1 Mice

The female BALB / c, C57BL / 6 and C3H / HeN mice were bred and maintained in the animal facility at Centro de Investigación y Estudios Avanzados (CINVESTAV). Animals were age (newborn to 8 weeks old) and sex matched.

### 4.2 Medium

Regular RPMI 1640 medium (Gibco, Grand Island, NY) supplemented with nonessential amino acids (Gibco), 5 × 10^– 5^ M 2-mercaptoethanol (Sigma Chemical Co., St. Louis, MO), 1 mM sodium pyruvate (Sigma), 2 mM glutamine (Sigma), and 10 % (v / v) FCS (Gibco) was used in all lymphocyte cultures.

### 4.3 Surface staining

Spleen was removed from mice at different ages. Briefly, the spleen cells were obtained by Ficoll (Sigma) gradient separation, then washed, counted and directly labeled with monoclonal antibodies. Bone marrow, Peyer's patches and peritoneal cell suspensions were also made from adult mice, cells were washed, counted and directly labeled with monoclonal antibodies. The antibodies used were: anti-IgM-FITC, anti-IgM-PE, anti-CD38-FITC, anti-CD-38-PE, anti-CD-38-biotin, anti-IgD-FITC, anti-B220-FITC (all from Southern Biotechnology Associates, Inc. Birmingham, AL), anti-CD5-PE, anti-B220-PE, anti-HSA-FITC and as isotype controls rat IgG1 and IgG2a (all from Pharmingen, San Diego, CA), and FITC-, PE- and PerCP-labeled streptavidin (all from Becton Dickinson, San José, CA). All cells were analyzed in a FacSort cell sorter (Becton Dickinson).

### 4.4 Purification of B lymphocytes by MACS by positive selection

Separation of B and T cells was done using a "High Gradient Magnetic Cell Separation" with Variomacs system (Miltenyi). Briefly, the spleen cells were obtained by Ficoll (Sigma) gradient separation, then washed and directly labeled with magnetic anti-B220 microbeads (5 × 10^7^ cells / ml). After a 30-min incubation period at 4 °C, the suspension was passed through a MACS column (Miltenyi) mounted into the MACS separator, and then unlabeled cells passing through the column were collected. The column was washed, removed from the magnet and bound cells were washed out. To verify the efficiency of the separation method, collected cells were stained with anti-Thy-1.2-FITC / B220RB-PE and analyzed with a FacSort cell sorter (Becton Dickinson) (B220^+^ cells were ≥ 95 %).

### 4.5 Proliferation of B cells

For proliferation, 0.5 × 10^6^ / ml purified B cells were incubated in 96-well flat-bottom tissue culture plates in 200 μl of medium with the following monoclonal antibodies: B7.6, anti-mouse μ-chain 35, at 10 μg / ml; NIM-R5, anti-mouse CD38 9, at 20 μg / ml; 1C10, anti-mouse CD40 36, at 10 μg / ml or LPS at 50 μg / ml plus 10 U / ml of IL-4 (gift from M. Howard, DNAX Research Institute, Palo Alto, CA). All cells were incubated for 72 h at 37 °C and pulsed with [^3^H] thymidine during the last 4 h of the culture before being harvested and counted.
